# Supplementary material for: Analysis of PPARγ Signaling Activity in Psoriasis
Source: Int J Mol Sci. 2021 Aug 10;22(16):8603. doi: 10.3390/ijms22168603 (PMC8395241; doi:10.3390/ijms22168603)
Supplement: Supplementary file 1 [file ijms-22-08603-s001.zip › Supplemental materials_Analysis of PPARg signaling activity in psoriasis/Pathway models/Models images and html files/PPARG inhibits phenotypes assotiated with psoriasis/125248.html]

dermatitis


# Disease dermatitis

|  |  |
| --- | --- |
| URN | urn:agi-meshdis:Dermatitis |
| Total Entities | 40 |
| Connectivity | 3220 |
| Name | dermatitis |

---

|  |  |
| --- | --- |
| ChildConcepts | pustulosis |
|  | radiation dermatitis |
|  | Farber disease |
|  | acrodermatitis |
|  | application site dermatitis |
|  | eczema |
|  | Blau syndrome |
|  | bullous dermatosis |
|  | Malassezia dermatitis |
|  | digital dermatitis |
|  | injection site dermatitis |
|  | pigmented purpuric dermatosis |
|  | Majeed syndrome |
|  | nummular eczema |
|  | polymorphous light eruption |
|  | plant dermatitis |
|  | erythroderma |
|  | Wells syndrome |
|  | contact dermatitis |
|  | autoimmune progesterone dermatitis |
|  | cercarial dermatitis |
|  | dermatitis herpetiformis |
|  | atopic dermatitis |
|  | diaper dermatitis |
|  | chronic granulomatous disease |
|  | neurodermatitis |
|  | occupational dermatitis |
|  | panniculitis |
|  | seborrheic dermatitis |
|  | eczematous skin disease |
|  | irritant contact dermatitis |
|  | perioral dermatitis |
|  | perifolliculitis |
|  | Wiskott-Aldrich syndrome |
|  | actinic dermatitis |
|  | experimental dermatitis |
|  | subcorneal pustular dermatosis |
|  | periocular dermatitis |
|  | erosive pustular dermatosis of the scalp |
|  | interstitial granulomatous dermatitis |

---

|  |  |
| --- | --- |
| Pathway | Apoptotic Keratinocytes Clearance Recession in Systemic Lupus Erythematosus |
|  | PPARG inhibits phenotypes assotiated with psoriasis |
|  | efects selected |
|  | Neighbors of dermatitis |

---

|  |  |
| --- | --- |
| MedScan ID | 9010183 |

---

|  |  |
| --- | --- |
| Alias | dermitis |
|  | Dermatitides |
|  | dermal inflammation |
|  | dermatitis |
|  | cutaneous inflammation |
|  | Skin Inflammations |
|  | cutaneous inflammations |
|  | Inflammation of the Skin |
|  | inflammations of the skin |
|  | dermal inflammations |
|  | inflammatory skin disease |
|  | inflammatory skin diseases |
|  | Skin Inflammation |

---

|  |  |
| --- | --- |
| MeSH ID | D003872 |
|  | M0006046 |

---

|  |  |
| --- | --- |
| MeSH Heading | Dermatitis |

---
